# Electronic Raman Scattering On Individual Semiconducting Single Walled Carbon Nanotubes

**DOI:** 10.1038/srep05969

**Published:** 2014-08-06

**Authors:** Xi Chen, Bairen Zhu, Anmin Zhang, Hualing Zeng, Qingming Zhang, Xiaodong Cui

**Affiliations:** 1 Department of Physics, The University of Hong Kong, Hong Kong, China; 2 Department of Physics and Beijing Key Laboratory of Opto-electronic Functional Materials & Micro-nano Devices, Renmin University of China, Beijing 100872, People's Republic of China; 3 Department of Physics, The Chinese University of Hong Kong, Hong Kong, China

## Abstract

We report experimental measurements of electronic Raman scattering by electrons (holes) in individual single-walled carbon nanotubes (SWNTs) under resonant conditions. The Raman scattering at low frequency range reveals a single particle excitation feature. And the dispersion of electronic structure around the center of Brillouin zone of a semiconducting SWNT (14, 13) is extracted.

Many experimental techniques aiming at charactering electronic properties surrender in intrinsic one-dimensional systems particularly single-walled carbon nanotubes (SWNTs) owing to their atomic size and richness of geometric structures. Typical examples include the magneto-electric transport techniques and angle-resolved photoemission spectroscopy which informatively probe the band dispersion and electron (hole)'s quantum states in 3D and 2D materials, but lose ground in SWNTs. As yet, there lacks a method capable of directly evaluating the band dispersion in SWNTs. Resonance Raman spectroscopy has been recognized as one of the most powerful and popular characterizing techniques in SWNT research. SWNTs' geometric structures could be quantitatively identified at single nanotube level with the well-established protocols in resonance Raman spectroscopy^1^. Intensive efforts in Raman spectroscopy have been focusing on the scattering by characteristic phonons. And the electronic aspects are implicitly addressed in Raman study by means of the dependence of scattering intensity on excitation energy, the energy shift due to electron-phonon coupling^2^, and the spectrum lineshape, for instance, Breit-Wigner-Fano (BWF) lineshape due to phonon-plasmon coupling and electron-electron interactions^1^,^3^,^4^.

Recently Farhat et al. reported a mode of electronic Raman scattering (ERS) from metallic SWNTs, where the Raman shift changes with the excitation energy and the scattered photon is exactly energetically resonant with the *M_ii_* excitonic transition energy^5^. The electric-doping modulation on the ERS shows that this mode of ERS originates from the electron-hole excitation by Coulomb exchange at the linear band of metallic SWNTs. However, the other ERS modes well established in conventional semiconductors, for example, the ones arising from single particle and collective elementary excitations which carry information on the electronic band dispersions, have not been observed yet.

There exist several obstacles toward the observation of ERS in SWNTs: (i) The scattered lights are overwhelmed by Rayleigh scatterings from the substrate and SWNTs, and inelastic scatterings by the lattice vibrations at finite temperatures. The intensity of the ERS is several orders of magnitude weaker than that of Rayleigh scatterings; (ii) The ERS originating from single particle and collective excitations is usually around a few meV in terms of energy shifted from the excitation laser. It is technically challenging to distinguish such a low frequency Raman signal from Rayleigh scattering; (iii) Resonance Raman scatterings from individual SWNTs in most experimental setups are conducted with a confocal-like micro-Raman setup in order to maximize the light collecting power, where the incident light and scattered light are both at normal direction with respect to the SWNT axis. The momentum of the incident and scattered photons are both orthogonal to that of electrons (holes) and therefore the photon and electronic elementary excitations are decoupled at normal incidence due to 1D nature of SWNTs. Here we report our observation of electronic Raman scattering around 1 meV from a small suspended semiconducting SWNT bundle. We study the low frequency Raman spectra on SWNTs under the resonant excitation at oblique incident angles. The energy of the ERS shows linearly proportional to the momentum exchange with the interacting photons along the direction of the SWNT axis. We attribute the ERS to the single-particle excitation, and the energy-momentum dispersion of the resonant band could be directly probed.

20 ~ 30 μm wide 1 mm long slits on silicon substrates were fabricated with a standard microelectromechanical (MEMS) process, including low pressure chemical vapor deposition (LPCVD) silicon nitride etching mask growth, optical lithography, reactive ion etching (RIE) and wet etching. The SWNTs were in situ synthesized by chemical vapor deposition (CVD) across the slits. The catalyst was prepared by selectively dipping the diluted solution of FeCl_3_ on the silicon substrate and then by being reduced under Ar/H_2_ 400 SCCM/50 SCCM at 900°C for 20 min. Individual SWNTs were grown on the substrates in ethanol vapor with the same gas mixture at 900°C for 1 h. For the Raman scattering experiments, the incident light from a single transverse mode HeNe laser (633 nm) was focused on SWNTs through a 10× objective at oblique angles against the SWNT axis and the scattered light was collected by a 50× objective, as sketched in Figure 1. The intensity of the incident light was kept below1 × 10^3^ W/cm^2^, and the acquisition time was 1800 s. The low frequency Raman signals was analyzed with a single stage monochromator (HR800, Jobin Yvon) with a set of Brag-Grate^TM^ notch filters optimized at anti-Stokes side which demonstrates much higher throughput than dual or triple stage monochromators, with high Rayleigh rejection rate at low frequency range^6^. Rayleigh scattering spectroscopy was carried out with a confocal like setup and a supercontinuum photonic fiber pumped by a nanosecond pulsed DPSS laser as the excitation source in a similar way as ref^7^,^8^.

Characteristic resonance Raman modes and Rayleigh scattering spectra are used to identify the sample's geometric structure, as demonstrated in Figure 2. The G-band Raman spectrum consisting of peaks of 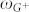 at 1591 cm^−1^ with linewidth 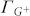 6 cm^−1^, as well as 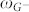 at 1559 cm^−1^, 1581 cm^−1^ and 1585 cm^−1^ indicates that the sample is a small bundle of semiconducting SWNTs^9^,^10^. From the empirical linear relation of *d* = 228/*ω_RBM_* (nm ·cm^−1^) between nanotube diameters and the inverse of their Radial Breathing Mode (RBM) frequencies of suspended SWNTs^11^, we further estimate the diameters of the SWNTs to be 1.8 nm (*ω_RBM_* = 125 cm^−1^) and 2.2 nm (*ω_RBM_* = 106 cm^−1^). The interband transition *S_ii_* is determined by Rayleigh scattering spectroscopy. As shown in Figure 2(b), the Lorentzian fitting 
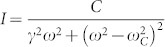
 of the Rayleigh spectrum yields two resonant energies of 1.91 eV and 1.87 eV respectively. According to the atlas of carbon nanotubes^12^ and the correction arising from the tube-tube interactions in bundles^13^, our samples are assigned to an ensemble consisting of a (14, 13) SWNT and a (23, 9) SWNT, where the redshifts by 20 meV and 50 meV owing to the intertube interactions are assumed respectively. The diameters of these two assigned SWNTs calculated by 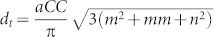 (*a_CC_* = 0.142 nm is the nearest-neighbor C-C distance)^11^ are consistent with those given by RBM Raman spectroscopy (Figure 2(a)). Consequently the peak around 1.91 eV in Rayleigh scattering spectrum shown in Figure 2(b) is assigned from the interband transition *S_33_* of SWNT with chiral indices (14, 13), while the one around 1.87 eV corresponds to the transition *S_44_* of nanotube (23,9).

Figure 3 shows the representative low frequency Raman scattering from the SWNT under resonant excitations at oblique incident angles. A small bump (Figure 3(a)) gradually rises from none at normal incidence to a few wave number (around 1 meV) at oblique incidence. The bump shows blue-shifted with the decrease of incident angle. The corresponding energy has a roughly linear dependence on *k_i_* cos *α*, the projection of the incident photon wave-vector along the SWNT axis, yielding the slope of 5.6 ~ 6.3 × 10^−5^ meV·cm. To our knowledge, the lowest frequency of phonon modes around *Γ*-point is in the range of 10 cm^−1^ ~ 12 cm^−1^^14^, significantly higher than that of the observed bump appearing in the range of 4.8 ~ 6.2 cm^−1^. Besides, the slope of the acoustic phonon dispersion near *Γ*-point is estimated to be 4 ~ 16 × 10^−7^ meV·cm^15^, which is two orders of magnitude smaller than our experimental observation. Therefore, possible origins associated with phonon dispersion can be ruled out. The energy of the small bump is independent of the excitation intensity in the range of 10^2^–10^3^ W/cm^2^, which modulates the effective carriers to some extent. Thus the bump unlikely originates from the Plasmon or other collective modes, as the corresponding dispersion is a monotonic function of effective carrier density but the observed features are independent of the excitation intensity. Besides, Rayleigh scattering implies that the contribution from SWNT (23, 9) can be ignored as the excitation energy (1.96 eV) is away from the resonance conditions at low frequency Raman energy. So we attribute the low frequency mode to the single particle excitation (SPE) of SWNT (14, 13), as illustrated in the inset of Figure 3(b).

Note that the hot electron-hole pairs generated in the optical transitions *S_ii_* relax and form excitons, a kind of quasiparticles in a time scale of picoseconds, while the ERS occurs almost instantaneously. So the ERS signal here reflects the properties of electrons (holes) instead of excitons. In the experimental setup, the incident angle concludes the photon's momentum projection *k_i_* cos *α* along the SWNT axis and therefore determines momentum transfer between the incident and scattered photons. As the requirements of energy conservation and momentum conservation along the SWNT axial direction, the energy difference between the incident and scattered photons exactly reflects the electronic band dispersion at resonance energy. Thanks to the perfect electron-hole symmetry for suspended SWNTs, the SPE simply probes the dispersion of the corresponding valence bands and conduction bands. This picture is qualitatively consistent with the fact that the Raman frequency increases with the increase of wave-vector transfer. As the magnitude of the wave-vector transfer is quite small (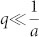), the quotient of 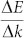 well presents the slope of the electronic band dispersion. Meanwhile the joint density of states of SWNTs follows 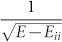 as a result of quasi 1D confinements, and consequently the electronic Raman process only occurs upon the resonance, namely around the inter-subband edge. Therefore, from the relation between Raman energy and the wave-vector transfer as plotted in Figure 3(b), we can estimate the slope of electronic energy band around the subband edges. A general linear behavior is also fitted in Figure 3(b), which yields an intercept of zero as expected from the band dispersion. The slope of the band dispersion at the 3rd subband edge (C_3_ and V_3_) is calculated to be in the range of 5.6 ~ 6.3 × 10^−7^ meV·cm with the axial wave-vector Δ*k* ranging from 9673 cm^−1^ to 13473 cm^−1^ in our experiments.

To qualitatively examine our experimental results, we apply the empirical formula^12^ below as the effective dispersion relation to estimate the slope of electronic band dispersion: 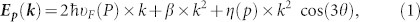
with 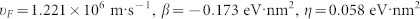 for S_33_ transition. Given the excitation of 1.96 eV (633 nm) in our experiments and the empirical dispersion, we derive the corresponding wave-vector on the electronic energy band of SWNT (14, 13) to be 1.446 × 10^7^ cm^−1^, and the slope at this point to be about 1 × 10^−4^ meV·cm. The calculated result shows a qualitative agreement with what we observed in the experiments. On the other hand, if we follow the model of exciton Kataura plots with environment corrections^16^,^17^, our sample would be assigned to a bundle composed of a (23, 1) nanotube and a (18, 11) nanotube. In T. Ando's theory^18^, the energy dispersion is described as 
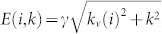
, where 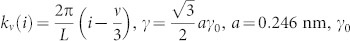 refers to the transfer integral between nearest-neighbor carbon atoms and is assumed to be 2.9 eV^19^. S_33_ transition of SWNT (23, 1) is now associated with the peak around 1.91 eV, so the slope 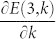 is calculated to be in the order of 2.5 × 10^−5^ meV·cm. This is qualitatively consistent with our experimental results, which provides a strong support for our explanation of SPE energy dispersion picture.

In summary, we report a low-frequency Raman mode from semiconducting SWNTs under resonant excitations at oblique incidence. The corresponding Raman mode shows a linear dependence on the momentum transfer along the SWNT. We attribute the new Raman mode to the inelastic scattering arising from electronic single particle excitation (SPE). The slope of electronic band dispersion at the subband edges is measured around 5.6 ~ 6.3 × 10^−7^ meV·cm on SWNT with structural index (14, 13).

## Author Contributions

X.D.C. conceived and designed the experiments; X.C., B.Z. and A.Z. conducted the experiments; X.C., B.Z., H.Z., Q.Z. and X.D.C. analyzed and interpreted the data; All authors discussed the results and wrote the manuscript.

## Figures and Tables

**Figure 1:**
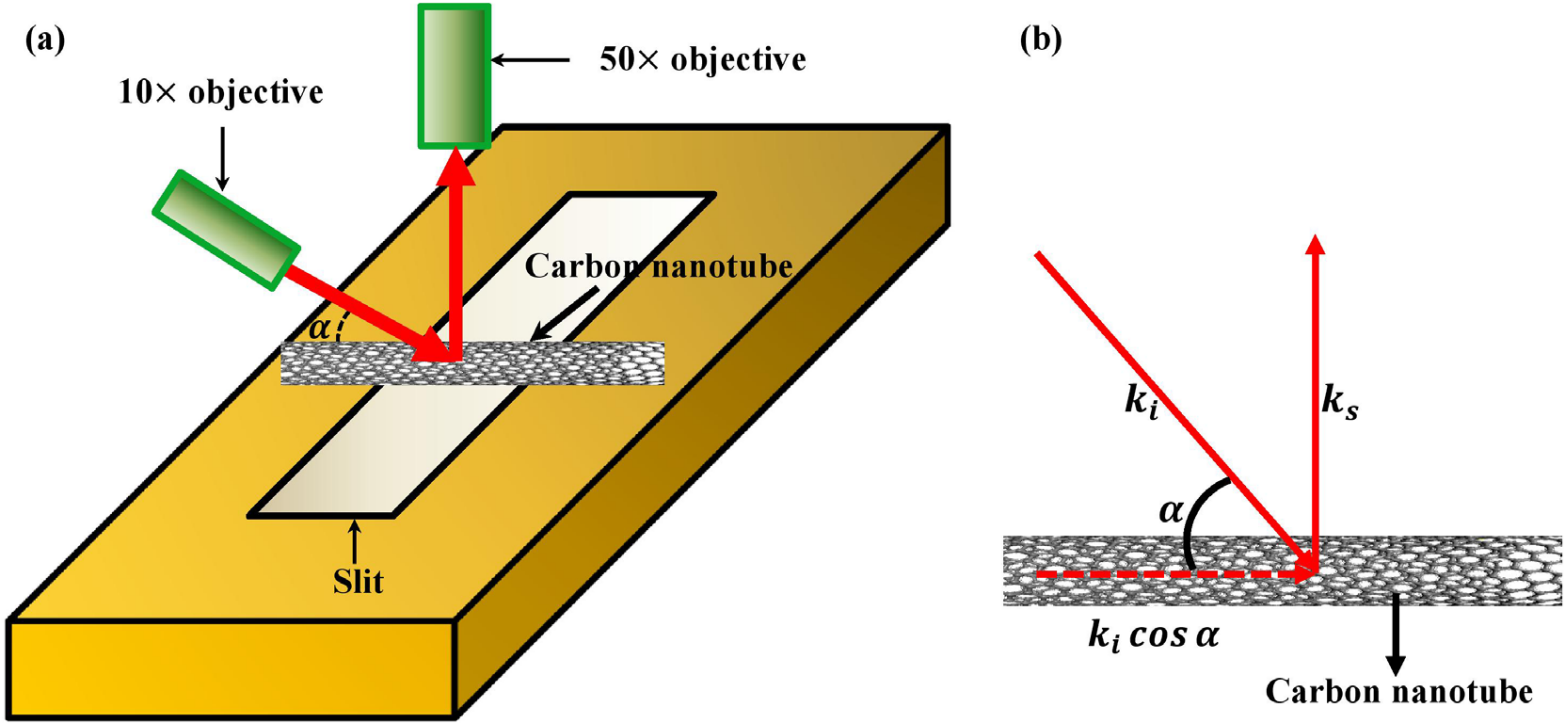
Schematic presentation of experimental geometry. (a) The excitation laser is shed at an oblique angle α against the SWNT suspended on the open slit of the substrate. The scattered light is collected at normal direction with respect to the SWNT axis. (b) Schematic of the scattering geometry with respect to the nanotube direction.

**Figure 2:**
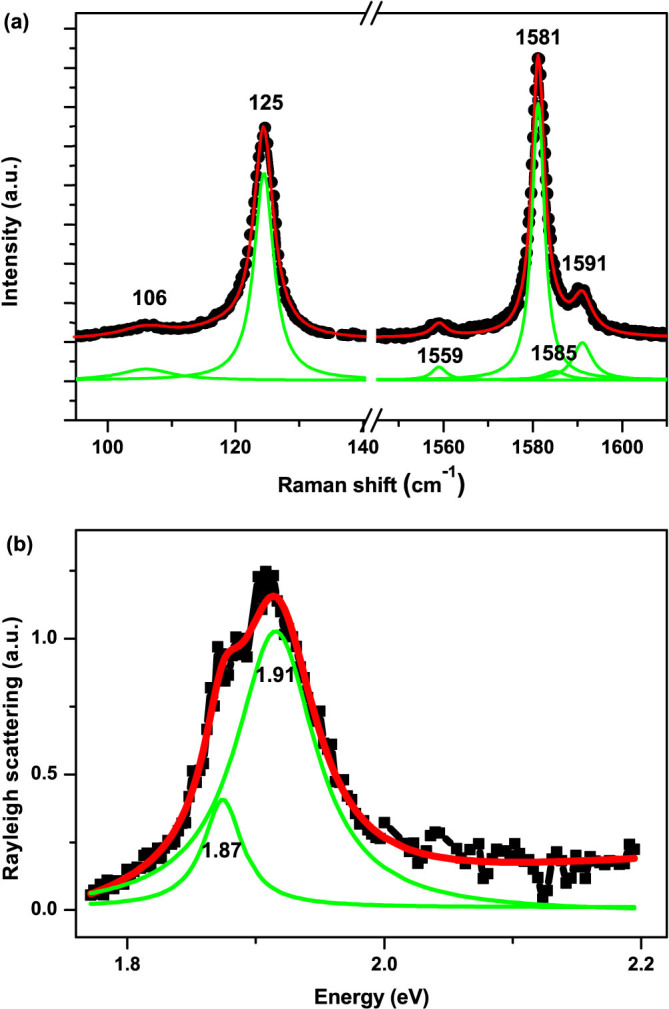
Characteristic resonance Raman spectra and Rayleigh scattering spectrum. (a) The RBM and G-band Raman spectra (black scattered lines). A superposition of multiple Lorentzian peaks (red curves) well describes the line shape. The individual Lorentzian peaks (green lines) (shifted vertically for clarity) imply a small SWNT bundle consisting of two semiconducting SWNTs with the diameters of 1.8 nm and 2.2 nm respectively. (b) Rayleigh scattering spectrum of the sample (black scattered line). A superposition of two Lorentzian fittings 
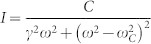
 (red curve) well describes the Rayleigh scattering, corresponding to *S_33_* transition of SWNT (14, 13) and S_44_ transition of SWNT (23, 9) according to the atlas of carbon nanotubes^12^ and the correction arising from the tube-tube interactions in bundles^13^.

**Figure 3:**
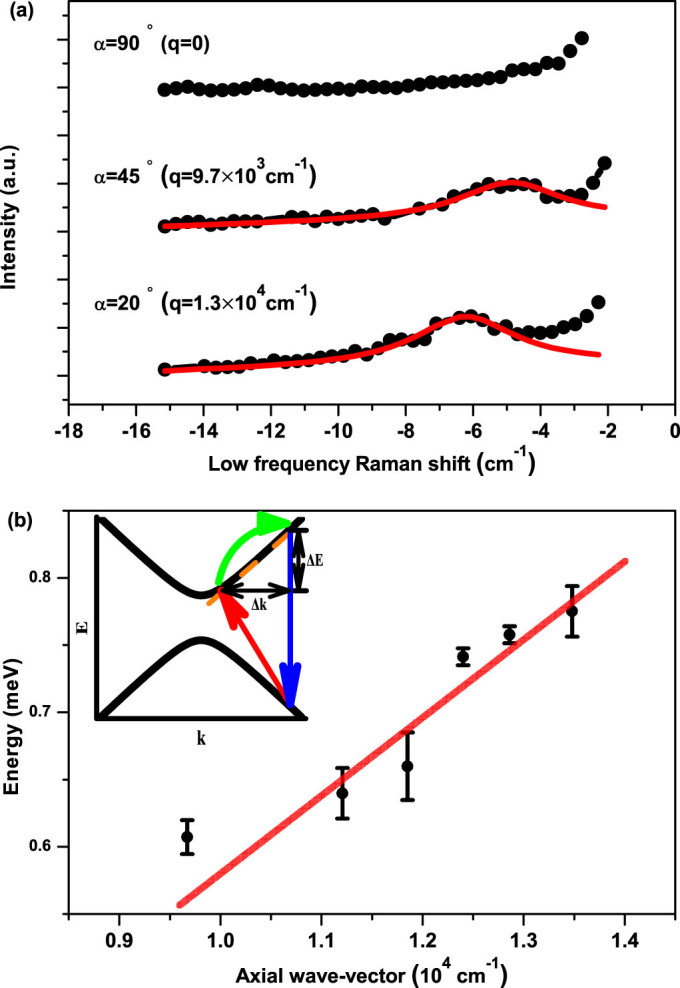
Electronic Raman spectra. (a) Representative Raman scattering (black scattered lines) at incident angle of 90°, 45°, 20° (q denotes the corresponding projection of the wave-vector along the SWNT axis at certain angle). The red lines following a Lorentzian lineshape are for highlight only. (b) Raman shift (in unit of energy) as a function of axial wave-vector (black scattered dots with error bars) exhibiting linear variations and the linear fitting (red straight line) of the data. Inset: schematic picture of the single particle excitation (SPE) model. The indirect transition denotes the optical interband transition with the momentum transfer from photons. The momentum transfer is disproportionally sketched to emphasize the non-zero projection of the wave-vector of photons along the SWNT axial direction.
